# Seropositivity of Hepatitis B and C among Syrian Multi-transfused Patients with Hemoglobinopathy

**DOI:** 10.4084/MJHID.2016.046

**Published:** 2016-09-01

**Authors:** Widad Yazaji, Wafa Habbal, Fawza Monem

**Affiliations:** 1 Department of Biochemistry and Microbiology, Faculty of Pharmacy, Damascus University, Damascus, Syria; 2 Clinical Laboratories Department, Al-Assad Hospital, Damascus University, PO Box 10769, Damascus, Syria

## Abstract

**Background and objectives:**

Blood transfusion is a lifesaving therapy for patients with hemoglobinopathies. However, the need of frequent transfusion carries the risk of transmitting hepatitis B and C infections which are intermediately prevalent in Syria. Despite screening blood donations with sensitive methods, the risk of transmission is still present when infectious blood is donated within the window period. This study aimed to investigate the incidence of HBV and HCV seropositivity, and its association with multiple transfusions among Syrian hemoglobinopathies patients.

**Materials and Methods:**

HBsAg, anti-HBc, anti-HBs and anti-HCV were tested for 159 Syrian multi-transfused patients by Enzyme-Linked Immunosorbent Assay (ELISA).

**Results:**

Thirty-nine of 159 (24.5%) multi-transfused patients were HBsAg/anti-HBc or anti-HCV positive, 26 (16%) of which never visited the dentist, and they either tested postsurgically negative for HBsAg and anti-HCV or never underwent a surgical procedure. On the contrary of anti-HCV seropositivity, HBsAg/anti-HBc seropositivity was significantly associated with the number of blood transfusions, number of blood units and age (P < 0.001).

**Conclusion:**

About one-sixth of our patients most likely acquired HBV/HCV infection via blood transfusion. Administering HBV vaccine, ensuring the immune status, and monitoring hepatitis markers might considerably minimize the incidence of viral hepatitis among multi-transfused patients.

## Introduction

Patients with hemoglobinopathies such as thalassemia, sickle cell anemia, and hemophilia need frequent and repeated blood transfusions to ensure their survival.^1^ Albeit an essential lifesaving intervention, blood transfusion carries the risk of transfusion-transmitted infections (TTIs), such as hepatitis B and C,^2^ which compose a major challenge to the public health worldwide.^3^ In general, screening blood donors with sensitive methods has reduced the incidence of TTIs.^4^ However, the risk of TTIs is still present when infectious blood is donated within the window period,^5^ especially among multi-transfused patients because of frequent exposure to blood products.^4^

In Syria, the risk is further augmented due to the intermediate prevalence of HBV and HCV among healthy blood donors.^6^–^8^ Moreover, since little information is available, healthcare provision to multi-transfused patients might lack hepatitis baseline assessment and follow-up. This study aimed to investigate the incidence of hepatitis B and C seropositivity, and its association with multiple transfusions among Syrian hemoglobinopathies patients.

## Materials and Methods

### Study population and sampling

This prospective cross-sectional study included 159 patients referred to the National Thalassemia Center, Ministry of Health or the Children Hospital, Damascus University in Damascus, Syria between October 2012 and December 2013. These patients underwent at least three repeated blood transfusions due to hemoglobinopathies, such as beta-thalassemia, sickle cell anemia, and sickle cell/beta-thalassemia disease, or hemophilia. Patients who had a hepatic infection, tattoo or a dentist visit during the previous three months before sampling were excluded.

Patients were interviewed, and their medical records were reviewed to obtain demographic data such as age, gender, marital status and occupation; medical data such as underlying diseases, surgical procedures, and hepatitis B vaccination status (vaccinated, three doses; unvaccinated, less than three doses; indeterminate, data not available); and transfusion history including number of both blood transfusions and units transfused to each patient.

Written informed consents were obtained from all adult patients or children guardians. Five milliliters of peripheral blood were collected and centrifuged at 2800 RCF for five minutes, and serum aliquots were kept at −80°C until the time of analysis. The study was approved by Damascus University Research Ethics Committee.

### Virological markers testing

Serological hepatitis B and C profiles (HBsAg, anti-HBc, anti-HBs and anti-HCV) were tested for each patient by Enzyme-Linked Immunosorbent Assay (ELISA; Biokit, Barcelona, Spain) and cut-off values were calculated according to manufacturer’s instructions.

HBsAg and/or anti-HBc positive sera and anti-HCV positive sera were considered for HBV DNA and HCV RNA testing, respectively. Viral nucleic acids were extracted using MagNA Pure Compact Nucleic Acid Isolation kit (Roche Diagnostics, Mannheim, Germany) according to the manufacturer’s instructions. HBV DNA and HCV RNA were detected by real-time PCR using LC-FastStart DNA Master HybProbe and LC-RNA Master HybProbe kits, respectively, on the LightCycler instrument (Roche Diagnostics, Mannheim, Germany) as previously described.^7^,^9^

### Statistical analysis

Data were presented as percentages and mean±SD. Chi-square and Fisher’s exact tests were used to study independence of variables using MicroSoft Excel 2007 and in-silico statistical calculator (http://in-silico.net/tools/statistics). Odds ratios were calculated to assess risk factors using MedCalc statistical software (http://www.medcalc.org/calc/odds_ratio.php). *P*-value < 0.05 was considered significant.

## Results

The study group included 88 males (55%) and 71 females (45%). The mean age was 17.11±9.25 years (range 1.5–52 years), and all patients were single. The underlying diagnosis denoted 124 patients with β-thalassemia, 21 with sickle cell/β-thalassemia, seven with sickle cell anemia, and seven with hemophilia. HBV vaccination status was designated as ‘vaccinated’ for 99 of 159 (62%), ‘unvaccinated’ for 24 of 159 (15%), and ‘indeterminate’ for 36 of 159 (23%) patients. None of the vaccinated patients received a booster dose. The mean numbers of blood transfusions and blood units were 146.08±100.1 (range 3–456) and 159.15±113.04 (range 3–563) per patient, respectively (Table 1).

Of 159 patients, one (0.63%), four (2.52%) and 84 (52.83%) patients were solely positive for HBsAg, anti-HBc and anti-HBs, respectively, and sixteen (10.06%) patients were concomitantly positive for anti-HBc and anti-HBs. Meanwhile, these markers tested all negative in the remaining 54 (33.96%) patients. On the other hand, eighteen (11.32%) patients were positive for anti-HCV which was undetectable in the remaining 141 (88.68%) patients. No co-seropositivity of HBsAg/anti-HBc and anti-HCV was observed. HCV RNA was detected in four of eighteen anti-HCV-positive patients, three of which were positive for anti-HBs alone, whereas HBV DNA was not detected in any of the 21 HBsAg/anti-HBc-positive patients.

None of the HBV/HCV seropositive patients reported a visit to the dentist except six HBsAg/anti-HBc seropositive patients and four anti-HCV and/or HCV RNA positive patients who visited the dentist 1.25 years (range five months-three years) and 1.6 years (range six months-three years), respectively, before sampling.

Thirteen of 21 HBsAg/anti-HBc seropositive patients underwent surgery, nine of which tested postsurgically negative for HBsAg. Six of eighteen anti-HCV seropositive patients underwent a surgery and tested all postsurgically negative for anti-HCV. The remaining eight and twelve HBsAg/anti-HBc and anti-HCV positive patients, respectively, never underwent any surgery (Table 2).

HBsAg and/or anti-HBc seropositivity was significantly associated with the number of blood transfusions (*P* = 0.03) and age (*P* < 0.001), but not associated with the number of blood units and gender (*P* > 0.05). Further analyses revealed an extremely significant association between HBsAg and/or anti-HBc seropositivity and the number of blood transfusions (*P* = 0.0006) and units (*P* = 0.0002) that exclusively ranged up to fifty and sixty transfusions/units, respectively. Anti-HCV seropositivity was significantly associated with gender (*P=* 0.02), but not associated with age and the number of blood transfusions/units (*P* > 0.05).

Patients older than seventeen years old and patients born before 1993 were at 0.16 (*P* = 0.0017) and 7.48 (*P*= 0.0006)-time-risk for acquiring HBsAg and/or anti-HBc seropositivity, respectively. Age was not a statistical risk factor for anti-HCV seropositivity (*P* > 0.05).

## Discussion

Syrian blood banks adhere to the WHO guidelines on assessing donors’ suitability and screening donated blood;^10^,^11^ i.e., they ensure that the donors, aged 18 to 65 years, are healthy with normal vital signs and hemoglobin level and weigh at least 50 kg. Donated blood units are screened for HBsAg, anti-HCV, HIV-1/2 antigens and antibodies, and anti-TP antibodies using recommended serological assays with a sensitivity and specificity, not less than 99.5%, such as enzyme immunoassay (EIA) and chemiluminescence microparticle immunoassay (CMIA). Finally, the ABO and Rh blood groups are typed including the weak D.

In Syria, a country of intermediate HBV and HCV prevalence,^6^–^8^ multitransfused patients represent a high-risk group to acquire HBV/HCV infection. Evidently, hepatitis seroprevalence among multitransfused patients is reflected by the seroprevalence among healthy blood donors.^12^ This is underlined by the consistency of our findings among multitransfused patients (HBsAg, 0.62%; anti-HBc, 12.5%) with a previous report on HBV seroprevalence among Syrian blood donors (HBsAg, 1.3%; anti-HBc, 11.2%,^7^ which coincides with other countries such as Jordan,^2^ Iran^1^,^13^ and Pakistan.^14^

Likewise, anti-HCV seropositivity in our patients (11.32%) was lower than those reported among multitransfused patients in Jordan (40%),^2^ Egypt (45% to 76%),^4^,^15^ Iran (44.7%),^16^ India (16% to 26%)^5^ and Pakistan (51.3%),^17^ which is reflected by the relatively lower prevalence (0.95%) of HCV seropositivity among Syrian blood donors.^8^

Our serological findings indicate exposure to either HBV or HCV in some of our patients. Although the route of transmission is not definitely asserted, chances of transmission can be presumed. Transmission via sexual contact or occupation is supposedly excluded since all our multitransfused patients had no sexual partner and none of them was a healthcare worker. Upon excluding thirteen patients who visited the dentist and/or underwent a surgery without postsurgical testing to negate hepatitis infection, twelve (7.5%) and fourteen (8.8%) of 159 patients most likely acquired HBV or HCV infection, respectively, via blood transfusion. Syrian blood banks execute WHO standards in screening blood donations, but the probability that some blood units elude the screening tests, especially if the donation occurs in the window period, cannot be excluded.

In our study, seropositivity of HBV was partially associated with the number of blood transfusions/units. Hence, each blood transfusion/unit composes a chance for acquiring TTI, and the more the blood transfusions/units, the higher the risk of exposure. However, such association seemed diluted by the coincidental nature of HBV transmission via blood transfusion. This is underlined by the significant association between our patients’ age and HBV seropositivity. Elder patients are relatively exposed to a longer-term risk^16^ and a higher number of transfused blood units due to their increased body weight. Moreover, the national program of HBV vaccination, launched in 1993, makes the younger patients at a lower risk to acquire HBV infection which coincides with other studies.^1^,^16^

Consequently, adopting a vaccination program for high-risk groups, such as multitransfused patients, is highly required. About one-third of our multitransfused patients was unvaccinated/indeterminate, and none of our vaccinated patients received a booster dose or had their anti-HBs levels monitored, which highlights the need for additional monitoring and prevention procedures. This is particularly essential for patients with hemoglobinopathies that require higher frequencies of blood transfusion.

On the other hand, low HCV prevalence in the Syrian population explains the lack of association between anti-HCV seropositivity and number of blood transfusions/units as well as age. This coincides with previous reports,^4^,^12^ but contradicts others.^3^,^14^,^16^–^18^ Gender does not seem a risk factor for acquiring TTI, but was unexpectedly associated with HCV seropositivity in our study; this finding was also reported elsewhere.^19^ Despite the lack of distinct explanation, this observation might be attributed to the impact of sex hormones on viral and host factors.^20^

## Conclusion

Despite the standard procedures followed by Syrian blood banks to ensure blood safety, patients with hemoglobinopathies are still at risk of acquiring HBV/HCV infection. Administering HBV vaccine, ensuring the immune status, and monitoring hepatitis markers might considerably minimize the incidence of viral hepatitis among multitransfused patients

## Figures and Tables

**Table 1:** Distribution of HBsAg/anti-HBc or anti-HCV positive multitransfused patients in terms of socio-demographic and medical characteristics (n=159).

Variable	Total No. (%)	HBsAg/anti-HBc positive No. (%)	*P*-value*	Anti-HCV positive No. (%)	*P*-value*
***Gender***					
**male**	88 (55%)	9 (5.6%)	0.2	10 (6.3%)	0.02
**female**	71 (45%)	12 (7.5%)		8 (5%)	

***Age***					
**≤17 year old**	86 (54%)	4 (2.5%)	0.0005	8 (5%)	0.38
**> 17 year old**	73 (46%)	17 (10.7%)		10 (6.3%)	

***Marital status***					
**with sexual partner**	0 (0%)	0 (0%)	NA	0 (0%)	NA
**without sexual partner**	159 (100%)	21 (13.2%)		18 (11.3%)	

***Occupation***					
**healthcare provider**	0 (0%)	0 (0%)	NA	0 (0%)	NA
**other**	159 (100%)	21 (13.2%)		18 (11.3%)	

***Underlying diagnosis***					
**β-thalassemia**	124 (78%)	16 (10%)	NA	13 (8.2%)	NA
**sickle cell β-thalassemia**	21 (13.2%)	4 (2.5%)		2 (1.25%)	
**sickle-cell anemia**	7 (4.4%)	1 (0.62%)		2 (1.25%)	
**hemophilia**	7 (4.4%)	0 (0%)		1 (0.62%)	

***Vaccination status***					
**vaccinated**	99 (62%)	13 (8.2%)	NA	NA	NA
**unvaccinated**	24 (15%)	3 (1.9%)			
**indeterminate**	36 (23%)	5 (3.1%)			

* *P*-value obtained by chi-square test using MicroSoft Excel 2007.

NA, non-applicable.

**Table 2:** Distribution of HBsAg/anti-HBc or anti-HCV positive multitransfused patients in terms of performing a surgery and/or visiting the dentist (n=39).

	Underwent a surgery n=19		Never underwent a surgery n=20
	HBsAg negative postsurgically n=15	HBsAg not tested postsurgically n=4	
**Dentist visited n=10**	HBsAg/anti-HBc positive n=3 Anti-HCV positive n=1	HBsAg/anti-HBc positive n=1 Anti-HCV positive n=0	HBsAg/anti-HBc positive n=2 Anti-HCV positive n=3
**Dentist not visited n=29**	HBsAg/anti-HBc positive n=6 Anti-HCV positive n=5	HBsAg/anti-HBc positive n=3 Anti-HCV positive n=0	HBsAg/anti-HBc positive n=6 Anti-HCV positive n=9
